# An open cluster-randomized, 18-month trial to compare the effectiveness of educational outreach visits with usual guideline dissemination to improve family physician prescribing

**DOI:** 10.1186/1748-5908-9-10

**Published:** 2014-01-15

**Authors:** Daniel Pinto, Bruno Heleno, David S Rodrigues, Ana Luísa Papoila, Isabel Santos, Pedro A Caetano

**Affiliations:** 1 Nova Medical School (Faculdade de Ciências Médicas-Universidade Nova de Lisboa), CEDOC, GIAI, Campo dos Mártires da Pátria 130, 1169-056 Lisbon, Portugal; 2 The Research Unit for General Practice and Section of General Practice, Department of Public Health, University of Copenhagen, Øster Farimagsgade 5, Postbox 2099, 1014 Copenhagen K, Denmark

**Keywords:** Educational outreach, Academic detailing, Guideline adherence, Family practice, Drug utilization, Program evaluation, Cost-benefit analysis

## Abstract

**Background:**

The Portuguese National Health Directorate has issued clinical practice guidelines on prescription of anti-inflammatory drugs, acid suppressive therapy, and antiplatelets. However, their effectiveness in changing actual practice is unknown.

**Methods:**

The study will compare the effectiveness of educational outreach visits regarding the improvement of compliance with clinical guidelines in primary care against usual dissemination strategies. A cost-benefit analysis will also be conducted. We will carry out a parallel, open, superiority, randomized trial directed to primary care physicians. Physicians will be recruited and allocated at a cluster-level (primary care unit) by minimization. Data will be analyzed at the physician level. Primary care units will be eligible if they use electronic prescribing and have at least four physicians willing to participate. Physicians in intervention units will be offered individual educational outreach visits (one for each guideline) at their workplace during a six-month period. Physicians in the control group will be offered a single unrelated group training session. Primary outcomes will be the proportion of cyclooxygenase-2 inhibitors prescribed in the anti-inflammatory class, and the proportion of omeprazole in the proton pump inhibitors class at 18 months post-intervention. Prescription data will be collected from the regional pharmacy claims database. We estimated a sample size of 110 physicians in each group, corresponding to 19 clusters with a mean size of 6 physicians. Outcome collection and data analysis will be blinded to allocation, but due to the nature of the intervention, physicians and detailers cannot be blinded.

**Discussion:**

This trial will attempt to address unresolved issues in the literature, namely, long term persistence of effect, the importance of sequential visits in an outreach program, and cost issues. If successful, this trial may be the cornerstone for deploying large scale educational outreach programs within the Portuguese National Health Service.

**Trial registration:**

ClinicalTrials.gov number NCT01984034.

## Background

Patients often do not receive treatment that is supported by best evidence. This includes both failure to provide treatment proven to be cost-effective and provision of care that is unnecessary or harmful [1]. High quality clinical guidelines synthesize the current best knowledge, make transparent recommendations for current best practice, and can improve the quality of care [2]. However, it is recognized that guidelines alone are insufficient to change clinical practice and that implementation strategies are required [3-5].

There is a wide range of such strategies but limited evidence to assess their comparative effectiveness, as there are few head-to-head trials. Some overviews of systematic reviews provide narrative synthesis of the evidence supporting the different interventions [1,4,6,7]. However, primary studies are too diverse and heterogeneous to allow for more robust methods of indirect comparison. The Cochrane Collaboration Effective Practice and Organization of Care Group has assessed several strategies through high quality systematic reviews. Printed educational materials have no apparent effect on processes of care, while educational meetings, educational outreach, local opinion leaders, audit and feedback, computerized reminders, and tailored interventions are associated with small but clinically significant improvements [8-14].

Educational outreach interventions are personal visits by a trained individual (hereafter named as detailer) to health professionals in their own settings [10]. This detailer is usually a healthcare professional (physician, nurse or pharmacist) with special training in communication skills. He or she presents educational contents prepared by an independent organization (such as a university) to an individual physician. The Cochrane Review estimates a small but consistent effect on prescription improvement (median 4.8%, interquartile range 3.0% to 6.5%) [10].

### Local context

In Portugal, healthcare is provided by two overlapping systems: a publicly funded National Health Service (NHS), and voluntary private and public health insurance. The NHS has universal coverage, and 20% of the population has additional insurance coverage [15]. Thus, most primary care is provided by the NHS.

NHS Primary care physicians work in units typically with 4 to 12 doctors, along with nurses and secretaries. On average, each family physician cares for about 1,700 patients. The NHS distinguishes two types of primary care units. The default one is the ‘personalized care units’ model, in which professionals receive a fixed salary. The other model is the ‘family health units’, which enjoy greater functional and organizational autonomy [16]. ‘Family health units’ start as type-A units, in which professionals receive a fixed salary as in the former model. If these A units meet quality indicators targets, they become type-B units, in which health professionals have a mixed payment scheme that includes salary, capitation, and pay for performance.

Within this context, prescription drugs have a variable patient co-payment, depending on their therapeutic value [15]. Electronic prescribing has been mandatory for all NHS reimbursed drugs since 2012. All prescription information is collected centrally by NHS [17].

National prescribing guidelines are commissioned by the National Health Directorate (a government agency) to academic researchers and key opinion leaders. This agency also monitors the quality indicators set in each of its guidelines [18,19]. These guidelines are published in the agency’s website (http://www.dgs.pt). Health professionals are expected to visit this website regularly to keep up-to-date with the latest guidelines. Therefore, this study will not have a group of naive physicians unexposed to guidelines. For this reason, the control group is composed of physicians exposed to passive guideline dissemination (the usual implementation strategy).

### Choice of design

The design is a parallel, open, cluster, superiority randomized trial with a 1:1 allocation ratio. This study will assess the effect of educational outreach visits on physician compliance with prescription guidelines. Although the intervention is targeted at individual physicians, there is a risk of contamination if the randomization occurs at the individual level. This is because physicians in the same practice might be influenced by intervention subjects (*e.g.*, by raising awareness of the topics, through discussion of difficult patient cases, or by sharing visit content). Therefore, a cluster-randomized design is appropriate. Also, the costs (*e.g.*, travel expenses, salary costs of the detailers) in a cluster-randomized design will better approach the real costs of this intervention if implemented as a public health program. Therefore, to minimize contamination and for practical reasons, the unit of allocation will be the primary care unit. The unit of analysis will be the family physician.

### Aim and objectives

This trial aims to assess whether educational outreach visits are superior to usual implementation of guidelines regarding the reduction of inappropriate prescribing. The primary outcomes will be the long-term (18 months) effects in the prescription of cyclooxygenase-2 (COX-2) inhibitors, and omeprazole, by family physicians. The secondary outcomes will be the short (1 month) and medium-term (6 months) effects of educational outreach visits in the prescription of these two drug classes. Other secondary endpoints will be the short, medium and long-term effects of educational outreach visits in the prescription of clopidogrel. Finally, the trial will determine the cost-benefit of educational outreach visits.

## Methods

The study will be a parallel, cluster-randomized controlled trial comparing educational outreach visits with usual guideline implementation. Besides the standard description below, we also provide a summary of the intervention, a PaT plot, and a cascade diagram in Additional file 1[20,21]. This protocol was written in accordance with the Consolidated Standards of Reporting Trials (CONSORT) statement - Additional file 2.

### Participants and setting

The trial will recruit family physicians employed in primary care units of the National Health Service of the Lisbon region, Portugal. This region comprises over 3.5 million patients. A primary care unit (the cluster) will be eligible if it has at least four family physicians (which represent about 6,800 patients). Physicians that are planning to retire within two years, and those without an assigned or still building (far from the average number of patients) patient list will be excluded. At least four family physicians in each unit have to be willing to participate in order for the unit to be included in the trial.

**Figure 1:**
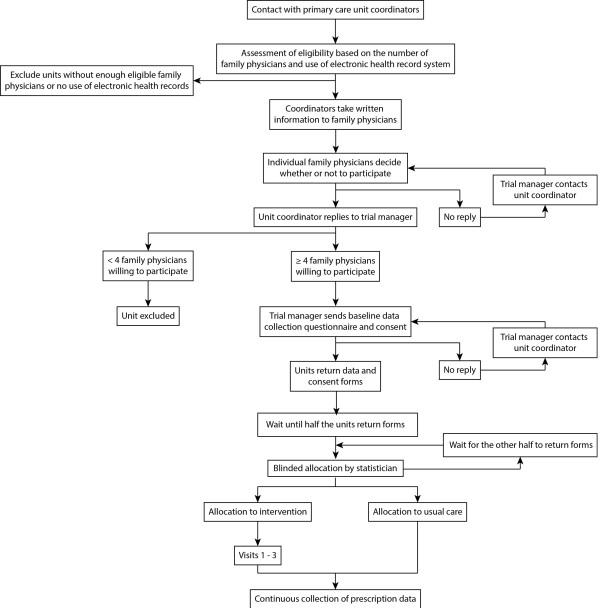
Flow of participants.

The expected participant flow is described in Figure 1. The research authors will meet with the coordinators of each unit, briefly explaining the protocol and extending them an invitation to participate. The coordinators will be asked to share trial information with other physicians in their units. The researchers will then contact and screen willing primary care units until the target number is met. The enrollment period will last six months. There will be no financial incentive for participation.

Baseline data will be collected from the primary care units (number of family physicians, type of primary care unit, urban versus rural setting, baseline prescription of COX-2 inhibitors, baseline prescription of omeprazole) and from the family physicians (age, gender, number of years of practice after vocational training, currently training residents). Participating physicians will agree to schedule three educational outreach visits, one for each guideline.

### Allocation to intervention and blinding

Clusters will be enrolled and allocated randomly. To achieve a good balance regarding baseline characteristics that can influence the outcome, the allocation sequence will be determined by minimization [22]. This stratified randomization method will balance the control and intervention groups for number of physicians (4 or 5, 6 or 7, 8 to 12), median baseline prescription of COX-2 inhibitors (above or below the regional median), median baseline prescription of omeprazole (above or below the regional median), proportion of physicians with fewer than 10 years of practice after completing vocational training (above or below 50%), and type of primary care unit (family health unit or personalized care unit). All physicians sending the consent statement before the cluster allocation will be included in the study.

The sequence of intervention visits for each unit will be determined by simple randomization.

Allocation concealment will be ensured by the following procedures: the trial manager (hired and not part of the authors research team) will assign a sequential number to each unit as participation forms are received; only anonymized data about participating units will be sent to the trial statistician (sequential number and minimization variables); data will be sent in two batches, one for each half of all units; the sequence of visits will be determined using the random sequence generator from Random.org (http://www.random.org/sequences/); the statistician will blindly allocate units to each trial arm using minimization and return allocation information to the trial manager.

Due to the nature of the intervention, neither family physicians nor detailers can be blinded. Outcomes are routinely collected by the regional health administration independently of the researchers or the trial and will only be sent to researchers after the intervention has ended. Upon receiving prescription data, the trial manager will generate a random code (using http://www.randomcodegenerator.com) to designate intervention and control units and another code for the order of the visits. Data analysts (the trial statistician plus one of the members of the research team) will receive a file with these codes, ensuring they will be blinded to group and visit sequence allocation until all analyses are completed.

### Intervention and comparison

Physicians in units randomized to the intervention will have three educational outreach visits during a six-month period. These visits will promote the implementation of governmental guidelines on the prescription of the following agents: non-steroidal anti-inflammatory drugs (NSAIDs) and COX-2 inhibitors, acid secretion modifiers, and antiplatelets [23-25]. The outcomes for the trial were chosen according to the main key-message from each guideline. For NSAIDs, COX-2 inhibitors should be prescribed only in patients with increased gastrointestinal risk who do not tolerate a classic NSAID associated with a gastroprotective agent; for acid secretion modifiers, all proton pump inhibitors are equivalent in effectiveness, so omeprazole should be used in most patients as it is the least expensive; for antiplatelets, there is no benefit of maintaining long term clopidogrel after a myocardial infarction, acute coronary syndrome, or percutaneous coronary intervention.

During each 15- to 20-minute visit, an academic detailer will promote one of the guidelines to a family doctor (up to three physicians may be present in each visit if they wish to, but one-to-one visits will be preferred and encouraged). The detailer will also distribute a point of care summary highlighting the main messages. The team of academic detailers will be as following: three members of the steering committee (two family physicians and one academic pharmacologist), three family physicians, and nine family medicine residents in the fourth and final year of their specialty training. The three members of the steering committee completed training in the methodology of academic detailing with the National Resource Center for Academic Detailing (Boston, MA). The other 12 detailers were trained locally by this steering committee, with pre-training study assignments, and 12 hours of face-to-face training that included the principles of academic detailing, role-playing, video-recording and feedback, discussion of the scientific content of each guideline, and knowledge assessment. To ensure consistency, the contents of each visit (structure, guideline features to highlight, and written materials) have been prepared in advance by the steering committee and were used in the training sessions. Whenever possible, a single detailer will perform all three visits to the same physician. The definition and methodology of educational outreach visits has been published elsewhere [10,26].

Usual guideline implementation consists of passive dissemination by their publication on the National Health Directorate’s website. Physicians in units randomized to the control group will be offered an unrelated training session (coding with the International Classification of Primary Care, second edition) as a token of good will for participating in the trial.

This trial has a pragmatic purpose. To improve adherence to the educational outreach visits, the Regional Health Administration will allow family physicians to use a patient visit slot (15 to 20 minutes) for each of the three visits, but physicians may also choose to have the visit before or after their regular hours. The first visit will be scheduled by the trial manager contacting the target family physician. Subsequent visits will be scheduled directly between the detailer and the target family physician. Also to improve adherence, participating physicians will be asked to allow us to use their personal phone numbers and emails. We will use these to send them a reminder of the visit two days before it is scheduled. Rescheduling the visit will be allowed up to the day before it is scheduled to take place; if the physician is unable to attend the visit but cannot warn the detailer beforehand, no additional efforts will be made to reschedule that visit (*i.e.*, the program will continue with the next guideline). The intervention will be discontinued at physician request or if the physician changes workplace. Participating physicians will not be prohibited from receiving any other interventions during the trial.

### Outcomes

#### Primary and secondary outcomes

There are two primary outcomes, measured at the physician’s level. One is the proportion of COX-2 inhibitors (anatomical therapeutic classification [ATC] M01AH) prescribed within the entire NSAID class (ATC M01A) in defined daily doses 18 months after the intervention. The other is the proportion of omeprazole (ATC A02BC01) within the entire proton pump inhibitors class (ATC A02BC) in defined daily doses 18 months after the intervention.

There are seven secondary outcomes, also measured at the physician’s level: the proportion of COX-2 inhibitors within the NSAID class at one and six months; the proportion of omeprazole within the proton pump inhibitors class at one and six months; and the number of defined daily doses of clopidogrel prescribed per 1,000 registered patients at 1, 6 and 18 months. The proportion of clopidogrel (ATC B01AC04) within the platelet aggregation inhibitors (ATC B01AC) was not selected as an outcome because the most commonly used drug in that class is acetylsalicylic acid (ATC B01AC06). This drug is generally sold over the counter, and no reliable data of its consumption exists.

#### Timing of outcome collection

Unlike a randomized controlled trial for a drug, we do not expect the intervention to be delivered immediately after allocation. This is because there are a limited number of detailers, and their time for visits is also limited (as they themselves are practicing physicians). Plus, family physicians’ availability to receive visits from these detailers is also constrained by their heavy workload. These constraints will prevent us from delivering the intervention to all physicians in a short period (*e.g.*, less than one month). We plan to deliver the full intervention to all physicians in the experimental group over a period of six months. For each guideline, we will seek to visit all the physicians belonging to the same cluster in the shortest time possible, to limit contamination within clusters. This will cause the gap between allocation and intervention dates to vary depending on the participating unit. Thus, if we assess the outcome at a fixed time following randomization, participating units will have different follow-up times after the intervention, and as a result the observed intervention effect will be unreliable. Moreover, since we will have three visits for each physician, there will also be differences in the time of intervention between guidelines. For these reasons, outcomes will be measured from specific intervention dates rather than from the general allocation date. This will provide more reliable data about the efficacy of the intervention.

Using the intervention dates poses a problem for the control group, for whom these do not exist. If we were to measure outcomes from the allocation date, there would be up to a six-month time gap compared with intervention units. In this relatively long interval, other factors influencing prescription of the study drugs could arise (*e.g.*, new research or guidelines being published, changes in drug policy in the Portuguese NHS, seasonal trends of prescription of NSAIDs, etc.). We will address this problem by randomizing the dates from which we measure outcomes for each guideline among control units. This will distribute control units along the intervention period, making them more comparable to the experimental group. This will be done by selecting a random month falling within the first and last months of the visits in the intervention group. The trial statistician will blindly assign a random month for each guideline in every control unit after the final visit in the intervention group is made and before outcomes are collected.

Outcomes will be measured using the same monthly prescription data for all physicians within a given cluster.

### Cost analysis

Global prescription spending will be defined as the sum of the cost of all drug prescriptions of NSAIDs (ATC M01A), acid suppressive therapy (proton pump inhibitors ATC A02BC and their alternatives: H_2_-receptor antagonists ATC A02BA, antacids ATC A02A, misoprostol ATC A02BB01, and sucralfate ATC A02BX02), and antiplatelets (ATC B01AC), up to 18 months after the intervention. These costs will be compared with the amount spent training the detailers, preparing and printing educational materials, travel expenses to intervention units, payment of detailers, program coordination, and physician time spent with a detailer rather than with a patient. Costs will be analyzed from the point of view of an educational outreach program rather than from conducting research. Therefore, research related costs (such as researcher time for data collection and analysis) will not be considered. Similarly, the unrelated training session offered to the control units will not be accounted for because it is only intended to improve recruitment and would not be necessary for the implementation of an educational outreach program.

### Data collection

Researchers will have access to prescription data through a data monitoring system operated by the Regional Health Administration. Data will be collected and provided by employees from this Administration according to researcher defined specifications. Importantly, researchers will not be directly involved in data collection. This information arrives with a two-month delay from the date the prescription is dispensed. The prescription information contained there can be either for acute conditions - single prescriptions with up to two packages to be dispensed within 30 days; or for chronic usage - three identical prescriptions (up to two packages each) to be dispensed within 6 months. Within the drug classes of this study, only NSAIDs cannot be prescribed for chronic usage. Adverse events cannot be collected in this study because only prescription data is available.

Detailers will record whether the planned visit was effectively accomplished, whether it had the planned duration, the number of physicians (including residents) present, whether the visit was made on patient visit time or off hours, the number of times the detailer had previously visited that physician, and feedback from the physician about the educational materials.

### Sample size

The research team has obtained pilot data from all physicians of three primary care units. This data was used to estimate within unit variability and the intra-cluster correlation coefficient (ICC). Data was also gathered for all the units in the Regional Health Administration to estimate the mean prescription and standard deviation for the primary outcomes. The mean proportion of omeprazole dispensed was 54.0% of all PPIs (standard deviation, SD, 10.1%) and the ICC was 0.027. The mean proportion of COX-2 inhibitors dispensed was 20.6% of all oral NSAIDs (SD 7.4%), and the ICC was 0.249. The Cochrane review on educational outreach visits found a median adjusted risk difference for improvement in compliance with desired practice of 5.6% (interquartile range 3.0% to 9.0%) and 4.8% specifically for prescribing (interquartile range 3.0% to 6.5%) in previous trials [10]. Therefore, we chose to calculate our sample size assuming the intervention would lead to a 5% absolute difference in compliance with guidelines between intervention and control units.

If we assume a mean cluster size of six physicians per unit, a 1:1 allocation ratio of controls per intervention unit, an alpha type error of 0.025, and a dropout rate of about 15%, then a sample of 110 physicians in each group will allow for 80% power to demonstrate a 5% absolute increase in the proportion omeprazole and a 5% absolute decrease in COX-2 inhibitors. To recruit the necessary number of physicians, 38 primary care units will be required. STATA 12.0 (STATA Corp, TX, USA) and its sampsi and sampclus commands were used to calculate sample size.

### Statistical methods

Physicians will be analyzed according to their randomly allocated group regardless of adherence to the intervention (intention to treat analysis). If physicians transfer to another unit within the health region, we will still be able to monitor their prescriptions. If the transfer is to a different health region (*i.e.*, not Lisbon) we will contact the physician and ask for the missing prescription data. In both cases, prescription of clopidogrel will be adjusted to the new patient list. If a physician retires or we are unable to retrieve data from a physician who moved to a different region, then we will use the last working month’s prescription.

Both groups will be compared on primary outcomes using generalized mixed-effects models. The ratio of COX-2 inhibitors to the entire NSAID class and the ratio of omeprazole to the entire proton pump inhibitor class and respective 95% confidence intervals will be calculated. Statistical significance will be assumed for a p-value less than 0.025. STATA 12.0 (STATA Corp, TX, USA) will be used to conduct the analysis.

### Data monitoring

Given the nature of the intervention, which poses minimal risks to patients, no data monitoring committee will be established.

### Ethical approval

This protocol has been approved by the ethics committee of the Regional Health Administration of Lisbon and Tagus Valley. Family physicians invited to participate will receive written information about the main aspects of the trial, namely which data are being collected and the procedures to ensure the non-identifiability of individual prescriber data. They will sign a written consent for researchers to access their data. The trial will only collect aggregated and non-identifiable patient data. As such, the ethics committee waived patient informed consent.

### Trial status

At the time of protocol submission, we have obtained ethical approval and have started to contact eligible primary care units. No primary care unit has been randomized.

The trial has been registered in ClinicalTrials.gov (NCT01984034) and ENCePP.eu (http://www.encepp.eu/encepp/viewResource.htm?id=5150).

## Discussion

### Strengths and limitations

Our pilot data about prescription of NSAIDs, acid-suppressive therapy, and antiplatelet therapy suggests that there is room for improving physicians’ prescribing, aligning it with evidence, and potentially leading both to improvements in patient outcomes and cost savings to the Portuguese National Health Service. This paper describes a protocol for a cluster-randomized trial to assess whether educational outreach visits have a long-term (18 months) effect on physician prescriptions. Randomized trials are the gold standard to assess intervention effects, and cluster-randomized trials are an appropriate design when the intervention is an education intervention targeted at healthcare providers [27].

In this trial, it will be impossible to blind physicians and detailers to the intervention. Lack of blinding is expected to overestimate the intervention effects [28,29]. To minimize the effect of this bias, we are using prescription, which is an objective outcome measured independently from the researchers. This outcome also minimizes attrition, since it is possible to continue to assess the prescription behavior even if a physician changes workplace (within the same region).

We have chosen prescription-related outcomes over clinical outcomes. In Portugal, hospital discharge diagnoses (coded through the 9^th^ revision of the International Classification of Diseases) are routinely collected for hospital reimbursement purposes, but are only available after considerable delay. Primary care diagnoses (coded through the 2^nd^ edition of the International Classification of Primary Care) began to be collected in 2007 and are available in the regional database within only one month of registration in the medical record. However, both hospital and primary care diagnoses have not been validated for comprehensiveness and accuracy. Moreover, diagnoses associated with incentives may be recorded more often than those without incentives [30,31]. Hence, we have chosen to use prescription patterns as main outcomes. All prescription data in the National Health Service is gathered by the Ministry of Health for pharmacy reimbursement purposes. Data are available for both ordered and dispensed prescriptions. Pilot data from three primary care units showed that only about 60% of the prescribed drugs are actually dispensed. This likely arises from several factors: lack of patient adherence to prescriptions, the inability of physicians to match monthly amounts of different medicines in the same prescription sheet (hence the patient will not fill the entire prescription), and errors in printing and composing prescriptions that are subsequently not handed to the patient nor removed from the electronic medical record. For all these reasons, we opted to use dispensed prescriptions data as primary outcomes rather than ordered prescriptions.

Proton pump inhibitors and antiplatelets may be placed in chronic prescriptions, valid for up to six months. This means that data for these drugs may include prescriptions as old as six months prior to dispensing (and hence up to six months prior to the intervention). This may result in an underestimation of the intervention’s effect in secondary outcomes (one and six months, which may still include many lingering old prescriptions). This underestimation will be resolved for the primary outcomes (18 months) because by that time all prescriptions issued prior to the intervention will have been dispensed. However, if the intervention effect decreases over time, prescriptions issued as early as 12 months after the intervention may still be dispensed at 18 months and thus lead to an overestimation of the primary outcomes.

The intervention’s effect on physicians may change over time. As the program moves forward, detailers are expected to build a relationship with visited physicians. This may improve the detailer’s ability to change the physician’s prescribing behavior. On the other hand, the physician’s curiosity and willingness to participate may decrease, making him or her less receptive to the intervention. To avoid confounding resulting from the order by which each guideline is presented, we will randomly assign the guideline order for each unit.

We are recruiting family physicians from the three types of primary care units described in the introduction. Preliminary data at the regional level suggests that prescription patterns have greater room for improvement in ‘personalized care units’ (COX-2 inhibitors market share within NSAIDs is 22.8% in personalized care units vs. 17.8% in family health units; and omeprazole market share within PPIs is 53.1% in personalized care units vs. 55.5% in family health units). However, it is possible that recruitment is stronger in type A and type B ‘family care units’, since their contracts with the Regional Health Administrations state incentives for participation in research projects and lower drug expenditure. This differential participation may compromise the generalizability of our findings to the Portuguese NHS, especially if physicians respond differently to educational outreach according to the type of unit in which they practice.

### Relationship to other studies and expected contribution of this trial

In 2008, a Cochrane Systematic review concluded that educational outreach visits had a small but consistent effect on improving prescribing behavior, and identified areas for further research, namely: head-to-head comparisons between different educational outreach strategies, process evaluation embedded into trials to assess which components influence the effectiveness of the intervention, inclusion of patient outcomes, measurement of costs and sustained/long term/multiple visits educational efforts [10]. Since its publication, a large number of trials of multifaceted interventions that included educational outreach visits have been published. However, it is not yet clear whether educational outreach has a sustained effect and whether the intervention is cost-effective in a broad range of healthcare systems. The current study is innovative and important internationally as it addresses both these questions. Locally, this trial may show the effectiveness and feasibility of a sustained educational outreach program. If successful, it may be the cornerstone for deploying large-scale programs within the Portuguese NHS. This may range from other types of prescription improvement (*e.g.*, much needed increase of generic market share, which in Portugal is only about half the share of Germany or the US; targeting innovative therapy for appropriate patients) to rational ordering of tests and even adequate screening practices. Ideally, governments and academic centers should be well positioned to apply our findings to a variety of educational outreach programs.

### Data sharing

All data is property of the Lisbon Regional Health Administration (Portuguese Ministry of Health). Other researchers wanting to access raw data will need to obtain authorization from this institution before data can be shared.

## Abbreviations

ATC: Anatomical therapeutic chemical classification; COX-2: Cyclooxygenase-2; ICC: Intra-cluster correlation coefficient; NHS: National Health Service [Portugal]; NSAIDs: Non-steroidal anti-inflammatory drugs; PPIs: Proton pump inhibitors; SD: Standard deviation.

## Competing interests

The authors declare they have no competing interests and no financial or non-financial conflicts of interest.

## Authors’ contributions

DP and PAC conceived the study. ALP, BH, DSR and IS contributed the study design. ALP, DP, DSR and PAC calculated sample size and planed the statistical analysis. PAC is the grant holder. DP, DSR, IS and PAC contributed to recruitment of participants, training of detailers and preparing educational content. All authors contributed to drafting the manuscript, refinement of the study protocol, and approval of the final manuscript.

## Authors’ information

DP: MD, PhD-candidate; Department of Family Medicine, Grupo de Investigação Académica Independente - Chronic Diseases Research Center; Faculdade de Ciências Médicas, Universidade Nova de Lisboa (NOVA Medical School).

BH: MD, PhD-candidate; Research Unit for General Practice and Section of General Practice, Department of Public Health, University of Copenhagen.

DSR: MD, MSc-candidate; Department of Family Medicine, Grupo de Investigação Académica Independente - Chronic Diseases Research Center; Faculdade de Ciências Médicas, Universidade Nova de Lisboa (NOVA Medical School).

ALP: BMath, MStat, PhD; Department of Biostatistics, Chronic Diseases Research Center; Faculdade de Ciências Médicas, Universidade Nova de Lisboa (NOVA Medical School).

IS: MD, MEd, PhD; Department of Family Medicine, Grupo de Investigação Académica Independente - Chronic Diseases Research Center; Faculdade de Ciências Médicas, Universidade Nova de Lisboa (NOVA Medical School).

PAC: PharmD, PhD, MPH; Department of Pharmacology, Grupo de Investigação Académica Independente - Chronic Diseases Research Center; Faculdade de Ciências Médicas, Universidade Nova de Lisboa (NOVA Medical School).

## Supplementary Material

Additional file 1 An open cluster-randomized, 18 month trial to compare the effectiveness of educational outreach visits with usual guideline dissemination to improve family physician prescribing. Click here for file

Additional file 2: Table S1 CONSORT 2010 checklist of information to include when reporting a cluster randomised trial. **Table S2.** Extension of CONSORT for abstracts 1, 2 to reports of cluster randomised trials. Click here for file
