# Supplementary material for: An open cluster-randomized, 18-month trial to compare the effectiveness of educational outreach visits with usual guideline dissemination to improve family physician prescribing
Source: Implement Sci. 2014 Jan 15;9:10. doi: 10.1186/1748-5908-9-10 (PMC4029170; doi:10.1186/1748-5908-9-10)
Supplement: Additional file 1 — An open cluster-randomized, 18 month trial to compare the effectiveness of educational outreach visits with usual guideline dissemination to improve family physician prescribing. [file 1748-5908-9-10-S1.pdf]

An open cluster-randomized, 18 month trial to compare the effectiveness of educational outreach visits with usual guideline dissemination to improve family physician prescribing – Appendix 1

Summary of the interventions:

a	**Trial setup**	
	a1	**Clinical practice guidelines development**<br>Prescribing guideline developed from the cultural adaptation of an existing international guideline, public discussion with medical specialists associations, and endorsement by Governmental Authorities and the Portuguese Medical Association.
	a2	Questionnaire with baseline characteristics of participating units: type of primary care unit, number of physicians, proportion of physicians with less than 10 years of practice.
	a3	Data collection from regional prescription registry: median prescription at baseline for COX-2 inhibitors and omeprazole for each of the primary care units willing to participate.
b	**Usual care: passive guideline dissemination**	
	b1	The three guidelines are published in the website of the National Health Directorate's (governmental authority) website.
	b2	Single group-based training session unrelated to prescription (coding with the International Classification of Primary Care, 2nd Edition)
	b3	For the control group the date for the "start" of intervention will be allocated randomly. These will be selected between the date of the first and last visit in the trial.
c	**Intervention arm: educational outreach visits**<br>An academically trained detailer will meet with family physicians (FP) in their practices, presenting the key messages of the guidelines and discussing barriers to guideline implementation. Individual visits will be encouraged, but up to three physicians may be present in each visit. There will be a total of three visits (one for each of the three guidelines) each lasting between 15 and 20 minutes. The order of the detailing visits will be randomised for each unit in the intervention arm.	
	c1	Training of academic detailers: Three members of the steering committee (2 FPs and 1 pharmacist) completed training in the methodology of academic detailing with the National Resource Center for Academic Detailing (Boston, MA). The other 12 detailers (3 FPs and 9 FP-trainees) were trained locally by the steering committee, with pre-training study assignments, and 12 hours of face-to-face training which included the principles of academic detailing, role-play, video-recording and feedback, discussion of the scientific content of each guideline, and knowledge assessment. To ensure consistency, the contents of each visit (structure, guideline features to highlight, and written materials) have been prepared in advanced by the steering committee and were used in the training sessions.
	c2	c2(1-3). Academic detailer delivers a detailing session. For each of the three guidelines, there will be a single detailing visit. Whenever possible, a single detailer will perform all visits to the same physician.
	c3	c3(1-3). The detailer will also distribute a point of care summary highlighting the main messages.
x	Interaction between physicians reinforce the intervention within a practice	
d	**Outcome collection**<br>I. Regional prescription registry extraction: COX-2 and NSAIDs prescription data will be collected at 1 month(d1), 6 months (d2) and 18 months (d3) after the respective educational visit has been performed (intervention group) or the equivalent randomly assigned visit date (control group).	

Timing of interventions and assessments (PaT plot)[1]

Time line	Educational outreach visit	Usual guideline dissemination
2-6 months pre-randomisation	a1 a2 a3<br>b1 c1	a1 a2 a3<br>b1
Allocation (minimization)		
During 6 months (0 ≤ t ≤ 6)	b1 c2.1 c3.1 x<br>b1 c2.2 c3.2 x<br>b1 c2.3 c3.3 x	b1 b2 x
1 month post-intervention (1 ≤ t ≤ 7)	b1<br>d1	b1<br>b3<br>d1
6 months post-intervention (6 ≤ t ≤ 12)	b1<br>d2	b1<br>d2
18 months post-intervention (24 ≤ t ≤ 30)	b1<br>d3	b1<br>d3

Plan for the delivery of the intervention (Cascade diagram)[2]

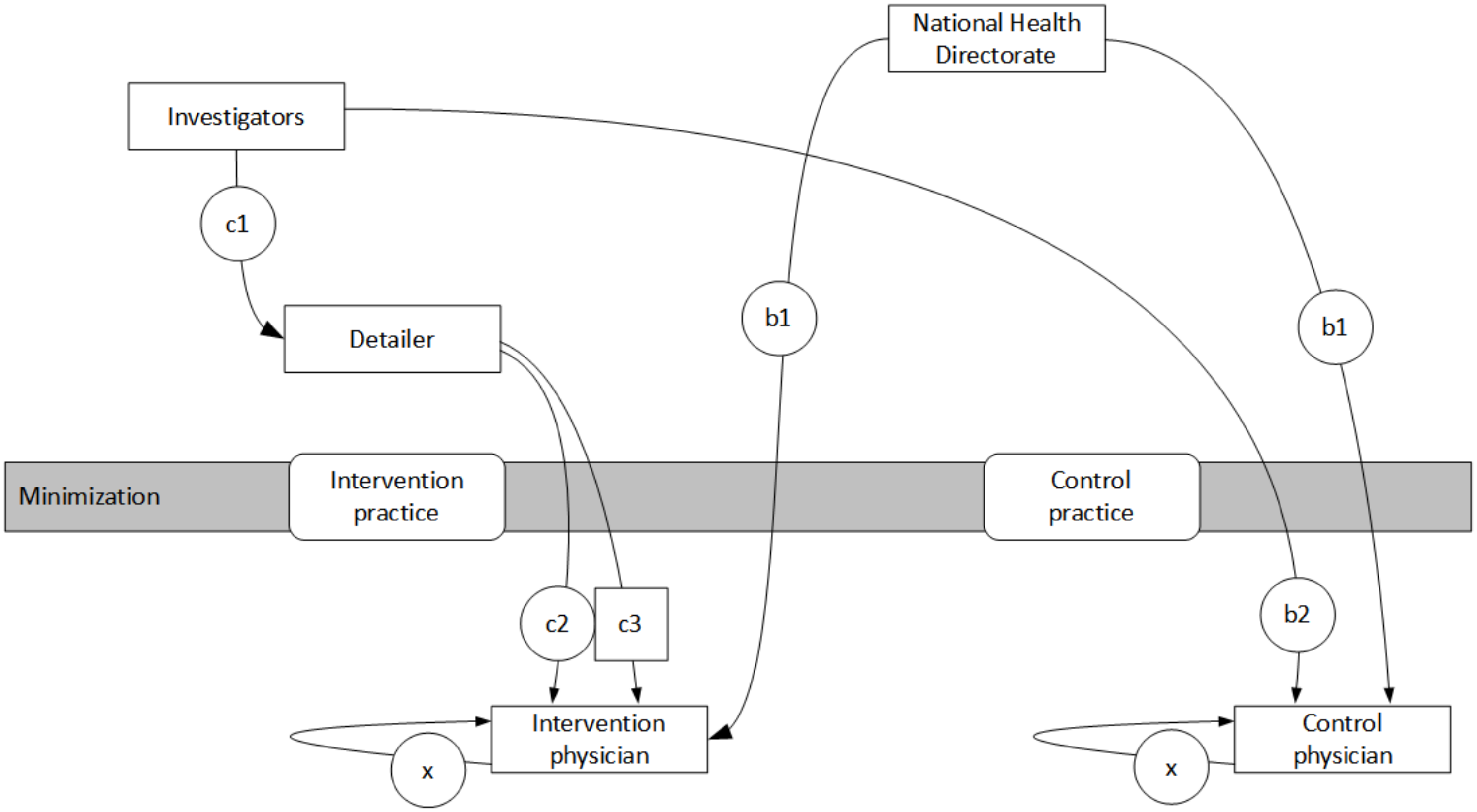

References

1. Perera R, Heneghan C, Yudkin P: **Graphical method for depicting randomised trials of complex interventions**. *BMJ* 2007, **334**:127–129.
2. Hooper R, Froud RJ, Bremner SA, Perera R, Eldridge S: **Cascade diagrams for depicting complex interventions in randomised trials**. *BMJ* 2013, **347**:f6681.
